# The relationship of nutritional risk with diet quality and health outcomes in community-dwelling older adults

**DOI:** 10.1007/s40520-021-01824-z

**Published:** 2021-07-13

**Authors:** Ilse Bloom, Anna Pilgrim, Karen A. Jameson, Elaine M. Dennison, Avan A. Sayer, Helen C. Roberts, Cyrus Cooper, Kate A. Ward, Sian M. Robinson

**Affiliations:** 1 MRC Lifecourse Epidemiology Unit, Southampton General Hospital, University of Southampton, Southampton, SO16 6YD UK; 2 NIHR Southampton Biomedical Research Centre, University of Southampton and University Hospital Southampton NHS Foundation Trust, Southampton, SO16 6YD UK; 3 NIHR Musculoskeletal Biomedical Research Unit, University of Oxford, Oxford, OX3 7LD UK; 4 AGE Research Group, Translational and Clinical Research Institute, Newcastle University, Newcastle upon Tyne, NE4 5PL UK; 5 NIHR Newcastle Biomedical Research Centre, Newcastle University and Newcastle Upon Tyne Hospitals NHS Foundation Trust, Newcastle upon Tyne, NE4 5PL UK; 6 Academic Geriatric Medicine, University of Southampton, Southampton, SO16 6YD UK; 7 NIHR Applied Research Collaboration (NIHR ARC) Wessex, University of Southampton, Southampton, SO16 7NP UK

**Keywords:** Community, Malnutrition, Nutritional risk, Older adults, Screening tool

## Abstract

**Objectives:**

To identify early nutritional risk in older populations, simple screening approaches are needed. This study aimed to compare nutrition risk scores, calculated from a short checklist, with diet quality and health outcomes, both at baseline and prospectively over a 2.5-year follow-up period; the association between baseline scores and risk of mortality over the follow-up period was assessed.

**Methods:**

The study included 86 community-dwelling older adults in Southampton, UK, recruited from outpatient clinics. At both assessments, hand grip strength was measured using a Jamar dynamometer. Diet was assessed using a short validated food frequency questionnaire; derived ‘prudent’ diet scores described diet quality. Body mass index (BMI) was calculated and weight loss was self-reported. Nutrition risk scores were calculated from a checklist adapted from the DETERMINE (range 0–17).

**Results:**

The mean age of participants at baseline (*n* = 86) was 78 (SD 8) years; half (53%) scored ‘moderate’ or ‘high’ nutritional risk, using the checklist adapted from DETERMINE. In cross-sectional analyses, after adjusting for age, sex and education, higher nutrition risk scores were associated with lower grip strength [difference in grip strength: − 0.09, 95% CI (− 0.17, − 0.02) SD per unit increase in nutrition risk score, *p* = 0.017] and poorer diet quality [prudent diet score: − 0.12, 95% CI (− 0.21, − 0.02) SD, *p* = 0.013]. The association with diet quality was robust to further adjustment for number of comorbidities, whereas the association with grip strength was attenuated. Nutrition risk scores were not related to reported weight loss or BMI at baseline. In longitudinal analyses there was an association between baseline nutrition risk score and lower grip strength at follow-up [fully-adjusted model: − 0.12, 95% CI (− 0.23, − 0.02) SD, *p* = 0.024]. Baseline nutrition risk score was also associated with greater risk of mortality [unadjusted hazard ratio per unit increase in score: 1.29 (1.01, 1.63), *p* = 0.039]; however, this association was attenuated after adjustment for sex and age.

**Conclusions:**

Cross-sectional associations between higher nutrition risk scores, assessed from a short checklist, and poorer diet quality suggest that this approach may hold promise as a simple way of screening older populations. Further larger prospective studies are needed to explore the predictive ability of this screening approach and its potential to detect nutritional risk in older adults.

## Introduction

The implementation of malnutrition screening, using standardised tools, has led to better recognition of the poorer health outcomes associated with it, such as sarcopenia, frailty and mortality [1–3]. Importantly, malnutrition is a common clinical problem in older populations [1]. Screening approaches that enable early identification of malnutrition risk in older people could be important to prevent the development of malnutrition, and the related detrimental effects on health [3]. Despite increased awareness of malnutrition, the preceding trajectories of change in dietary habits in older age, that can lead to greater nutritional risk, are poorly described [4]. Furthermore, as many malnutrition screening tools identify weight loss and thinness, they may not be designed to describe other aspects of poor nutrition, such as poor diet quality, low protein intakes, insufficient micronutrient intakes (such as vitamins D, E, C and folate) and micronutrient deficiencies (such as vitamin B12 and folate) and, therefore, lack sensitivity to identify those at risk [5, 6]. To identify signs of early nutritional risk, and to allow intervention before overt malnutrition develops, a different approach to screening is required. One such tool is the ‘Determine your Nutritional Health’ (DETERMINE) tool, developed by the US Nutrition Screening Initiative to identify and treat nutritional problems in older populations [7]. DETERMINE was designed for self-completion, requiring no specialist knowledge or equipment. The tool includes ten questions on age-related and contextual factors that are linked to poor nutrition in older age; responses are weighted to calculate an overall nutrition risk ‘score’, with thresholds given to identify categories of risk. Older adults with high nutritional risk, assessed using this tool, have been shown to be more likely to have low nutrient intakes and to report poorer health [7]. However, studies of its prediction of mortality in older populations have yielded mixed findings [8–10].

Therefore, the aim of the current study was to assess the use of an adapted DETERMINE checklist to calculate a nutrition risk score in a group of older community-dwelling adults in the UK. In this exploratory study, we assess the utility of this approach by determining associations of the score with hand grip strength, which is linked to malnutrition [11], diet quality, body mass index and reported weight loss, both cross-sectional and longitudinally over a 2.5-year period, and by evaluating the association between the baseline score and risk of mortality over the follow-up period.

## Methods

### Participants

At baseline, 86 older adults were recruited to the study from three outpatient clinics in Southampton [*n* = 27 (31%) Comprehensive Geriatric Assessment (CGA); *n* = 32 (37%) syncope clinic; *n* = 27 (31%) fragility fracture clinic]. A total of 545 patients from these clinics were screened for eligibility; of these, 224 were eligible to take part, and 86 (38%) agreed to participate in the study. Eligibility criteria were: aged 60 years or older, not acutely unwell, capable of giving informed consent. All participants who expressed an interest were visited at baseline at home by a researcher (AP), between March 2015 and June 2016. The participants were followed up by the same researcher (AP) 2.5 years after baseline, between September 2017 and December 2018. Of the 86 participants who were visited at baseline, 8 (9%) died during the follow-up period (with date of death available); 53 (62%) received a follow-up home visit; the remaining 25 (29%) participants were not followed up for the following reasons: death with no date available (*n* = 1), lacking capacity to consent (*n* = 5), declined (*n* = 15), relocated (*n* = 2), they were too unwell (*n* = 2).

The study had ethical approval from the National Research Ethics Service Committee Southwest, 14/SW/1129. All participants gave written informed consent. The datasets generated and analysed during the current study are available from the corresponding author on reasonable request.

### Home visits—baseline and follow-up

Information on background characteristics, including age and age at leaving full-time education, was obtained by questionnaire at the baseline interviews. At both interviews data were collected on lifestyle, health, social, and psychological factors [12–18]. Participants reported their number of doctor-diagnosed comorbidities out of the following: heart attack, congestive heart failure, angina, stroke, mini-stroke or transient ischemic attack (TIA), hypertension, diabetes, asthma, depression, chronic lung disease, kidney disease, cancer, or any other serious disease. Appetite was assessed using the Simplified Nutritional Appetite Questionnaire (SNAQ) [19]. Grip strength and weight were measured.

### Outcome measures assessed at baseline and follow-up

#### Grip strength

Maximal grip strength was measured using a handgrip Jamar dynamometer (Lafayette Instrument Company, USA). Grip strength was measured, to the nearest kg, three times in each hand and the maximum value was used for the analyses [20].

#### Diet quality

Diet was assessed using an administered 24-item food frequency questionnaire that was developed to describe diet quality in community-dwelling older adults [13]. Based on a participant’s reported frequencies of consumption of the listed foods, a ‘prudent’ dietary pattern score is calculated which describes compliance with this pattern [13]. High scores indicate diets characterised by frequent consumption of fruit, vegetables, wholegrain cereals and oily fish but low consumption of white bread, added sugar, full-fat dairy products, chips and processed meat, aligning with healthy eating guidance [13]. Participants’ prudent diet scores were interpreted as an indication of their diet quality.

#### Body mass index (BMI)

Self-reported height was recorded. Body weight was measured to the nearest 0.1 kg, with the participant wearing clothes and shoes, using portable SECA standing balance scales (model 875). BMI (kg/m^2^) was calculated [weight (kg)/height (m)^2^].

#### Weight loss

Weight loss was self-reported; participants were asked if they had lost any weight unintentionally within the past 12 months and, if so, how much weight they had lost to the nearest 0.1 kg.

#### Mortality

Participant deaths from any cause, from baseline until the follow-up home visit, were extracted from medical records.

### Nutrition risk score

A nutrition risk score was calculated for each participant at baseline (*n* = 86), and at follow-up (*n* = 53), using a checklist adapted from the DETERMINE checklist (Table 1 shows the original DETERMINE checklist) [7]. Our adapted checklist was based on eight of the ten components assessed in the DETERMINE; we omitted two items: 3 (‘I eat few fruits or vegetables or milk products’) and 9 (‘without wanting to, I have lost or gained 10 lb in the last 6 months) as diet quality and weight loss were outcome measures in our analyses.

**Table 1:** Original DETERMINE checklist showing the weighting and scoring used to derive a total nutrition risk score [7]

Item		Weighting
1	I have an illness or condition that made me change the kind and/or amount of food I eat	2
2	I eat fewer than 2 meals per day	3
3	I eat few fruits or vegetables or milk products	2
4	I have 3 or more drinks of beer, liquor or wine almost every day	2
5	I have tooth or mouth problems that make it hard for me to eat	2
6	I don’t always have enough money to buy the food I need	4
7	I eat alone most of the time	1
8	I take 3 or more different prescribed or over-the-counter drugs a day	1
9	Without wanting to, I have lost or gained 10 lb in the last 6 months	2
10	I am not always physically able to shop, cook and/or feed myself	2
Total score	0–2 = low nutritional risk 3–5 = moderate nutritional risk ≥ 6 = high nutritional risk	

For each ‘yes’ answer, the value in the weighting column is scored

We applied the published weighting scores to the remaining eight components in the checklist to calculate nutrition risk scores (Table 1). However, there were some differences in the way that variables were derived, when compared with the original study. In some cases these were differences in wording such that information collected from participants was mapped onto the original DETERMINE questions to derive equivalent information. For example, DETERMINE item 2 (‘I eat fewer than 2 meals per day’) was defined from participant responses to a question in the SNAQ [19] (‘Normally I eat < 1, 1, 2, 3, > 3 meals a day’; < 1 or 1 meal per day scored 3). In other cases, we used related background information to derive an equivalent variable. For example, we assessed food insecurity using a six-item food security module [21]; participants who were food insecure (score ≥ 2) were given a weighting of 4 (item 6, Table 1).

### Statistical analysis

Baseline descriptive characteristics are given as mean with standard deviation (SD) for continuous normally distributed variables, median with interquartile range (IQR) for continuous variables with a skewed distribution, or counts and percentages for categorical variables, as appropriate. The calculated nutrition risk score was used as a continuous variable in regression analyses, but shown in categories for presentation (‘low’ (0–2), ‘moderate’ (3–5) and ‘high’ (≥ 6) nutritional risk, Table 1); although we did not include two of the factors in our nutrition risk score calculations we used the published DETERMINE thresholds to categorise different levels of risk [7]. The relationships between nutrition risk score and grip strength, diet quality (prudent diet score) and BMI were examined using multivariate linear regressions. Since BMI was not normally distributed, a Fisher–Yates rank-based inverse normal transformation was performed to create *z*-scores (FY *z*-scores). We also transformed the prudent diet score and grip strength variables to create *z*-scores (FY *z*-scores) to enable the comparison of effect sizes. The association between nutrition risk score and weight loss (any unintentional weight loss in preceding year: yes/no), was examined using multivariate logistic regressions. Additional cross-sectional analyses considered whether inclusion of weight loss in the calculation of the nutrition risk score made a difference in terms of its associations with grip strength and diet quality. In the follow-up sub-group, longitudinal associations between baseline nutrition risk score and follow-up level of outcome measures were examined. As we have previously shown that prudent diet scores are generally higher among older women, compared with older men, and diet quality is positively associated with education [22], we adjusted for sex and education in our multivariate models. Analyses were performed with adjustments for sex, age and age left education; final models also took account of the number of comorbidities, type of clinic attended, and of follow-up time (in longitudinal analyses).

Additional analyses examined the relationship between baseline nutrition risk scores and risk of mortality in the period between baseline and follow-up home visits using Cox regression, with and without adjustment for age and sex. This was conducted among the 61 participants who either attended the follow-up home visit and were censored at this date (*n* = 53) or who died between the baseline and follow-up home visits with a date of death available (*n* = 8). Finally, receiver operating characteristic (ROC) analyses were performed to further evaluate the predictive value of nutrition risk scores in relation to low grip strength (both at baseline and at follow-up) (using the EWGSOP2 cut-off points of < 27 kg (men) and < 16 kg (women) [2]) and poor diet quality (both at baseline and at follow-up) (prudent diet scores in the lowest quarter of the distribution). Data were analysed using Stata version 14.2.

## Results

At baseline, participants (*n* = 86) were aged between 60 and 93 years (mean age 78 (SD 8) years) and 53 (62%) of the study participants were women. The baseline characteristics are shown for the whole group, and according to category of nutritional risk, in Table 2. Over a third (36%) of all participants were living alone. The median nutrition risk score at baseline was 3 (IQR 1–5). Almost half (*n* = 40, 47%) of the participants were in the low nutritional risk category, 31 (36%) were at moderate risk and 15 (17%) were at high risk. As there were no statistically significant differences in nutrition risk scores between men and women [median nutrition risk score: 2 (IQR 1–5) in men, and 3 (IQR 1–4) in women, *p* = 0.276], separate analyses were not carried out.

**Table 2:** Baseline characteristics of the study participants, for the whole group and according to nutritional risk category

	Nutritional risk												
	All			Low (score 0–2)			Moderate (score 3–5)			High (score ≥ 6)			*p* value^a^
	*N*	Mean	SD	*N*	Mean	SD	*N*	Mean	SD	*N*	Mean	SD	
Age (years)	86	77.5	8.3	40	74.0	7.0	31	80.3	8.3	15	81.4	7.7	< 0.001
Height (cm)	82	165.8	9.3	38	165.6	9.5	31	167.6	9.3	13	162.4	8.0	0.290
Number of comorbidities	86	4.4	2.4	40	4.0	2.3	31	4.4	2.4	15	5.7	2.4	< 0.001
Prudent diet score	84	0.63	1.61	38	0.95	1.52	31	0.69	1.80	15	-0.29	1.03	0.007
Grip strength (kg)	85	23.6	9.3	40	26.2	9.1	31	23.6	8.9	14	16.2	6.8	< 0.001

	Total *N*	*N*	%	Total *N*	*N*	%	Total *N*	*N*	%	Total *N*	*N*	%	*p* value^a^
Weight loss (unintentional weight loss in preceding year)	86			40			31			15			
No		64	74.4		30	75.0		24	77.4		10	66.7	0.460
Yes		22	25.6		10	25.0		7	22.6		5	33.3	

	*N*	Median	IQR	*N*	Median	IQR	*N*	Median	IQR	*N*	Median	IQR	*p* value^a^
Weight (kg)	78	70.7	62.6–85.0	37	75.0	63.4–84.4	29	67.2	59.8–87.4	12	70.7	66.9–82.0	0.663
BMI (kg/m^2^)	77	26.6	23.9–30.2	36	26.9	23.8–30.4	29	24.8	23.2–28.2	12	26.6	24.4–30.5	0.848

An adapted DETERMINE checklist was used to derive a nutrition risk score; participant characteristics are shown according to the published thresholds to categorise different levels of risk [7]

^a^Unadjusted *p* value for trend across the continuous nutrition risk score variable (values ranging from 0 to 11)

**Fig. 1:**
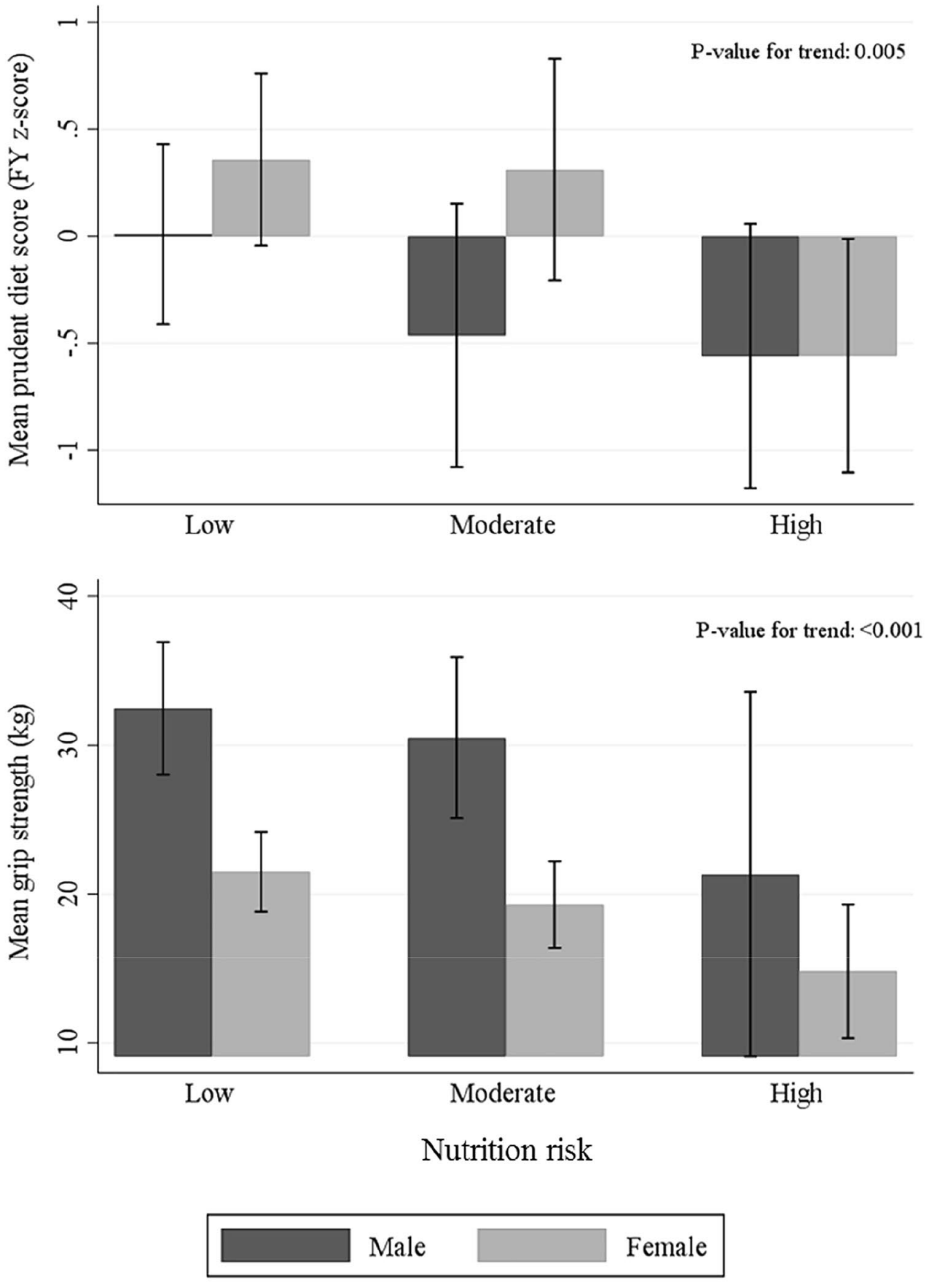
Diet quality [prudent diet score (*z*-score)] and grip strength (kg) according to category of nutritional risk at baseline [7], in older men and women studied (bars represent 95% CI for mean). Unadjusted *p* values for trend across the continuous nutrition risk score variable (values ranging from 0–11) among the pooled sample of men and women are shown

Participants with greater nutritional risk tended to be older and to have a greater number of comorbidities (Table 2). Univariate analyses showed a strong association between age and nutrition risk score at baseline (Table 2), such that 67% (*n* = 10) of the high risk group were aged over 80 years, compared with 23% (*n* = 9) of the low risk group. A higher nutrition risk score was associated with lower grip strength and poorer diet quality, but was not related to BMI or reported weight loss. The associations with diet quality and grip strength are illustrated in Fig. 1.

Table 3 shows the associations between baseline nutrition risk score and grip strength, prudent diet score, body mass index (BMI) and weight loss, in the multivariate models.

**Table 3:** Standard deviation difference in outcomes at baseline and follow-up per unit increase in baseline nutrition risk score

Outcomes	Adjusted for sex, age and age left education		Fully-adjusted^a^	
	Regression coefficient (95% CI)	*p* value	Regression coefficient (95% CI)	*p* value
**Outcomes at baseline**				
Grip strength (FY *z*-score)	**−** **0.09 (− 0.17, − 0.02)**	**0.017**	− 0.05 (− 0.13, 0.03)	0.207
Prudent diet score (FY *z*-score)	**− 0.12 (− 0.21, − 0.02)**	**0.013**	**− 0.11 (− 0.21, − 0.01)**	**0.032**
BMI (FY *z*-score)	0.08 (− 0.02, 0.18)	0.100	0.07 (− 0.05, 0.18)	0.245
Weight loss (odds ratios presented)	1.03 (0.83, 1.29)	0.785	1.01 (0.79, 1.28)	0.967
**Outcomes at follow-up**^b^				
Grip strength (FY *z*-score)	**− 0.15 (− 0.24, − 0.05)**	**0.003**	**− 0.12 (− 0.23, − 0.02)**	**0.024**
Prudent diet (FY *z*-score)	− 0.05 (− 0.18, 0.09)	0.482	0.02 (− 0.12, 0.16)	0.802
BMI (FY *z*-score)	**0.19 (0.04, 0.33)**	**0.012**	0.15 (− 0.01, 0.32)	0.068
Weight loss (odds ratios presented)	1.26 (0.88, 1.80)	0.205	1.40 (0.84, 2.32)	0.201

Significant associations (*p* < 0.05) are highlighted in bold

*CI* confidence interval, *FY* Fisher–Yates

^a^Adjusted for sex, age, age left education, no. of comorbidities (self-reported number of doctor-diagnosed comorbidities out of the following: heart attack, congestive heart failure, angina, stroke, mini-stroke or transient ischemic attack (TIA), hypertension, diabetes, asthma, depression, chronic lung disease, kidney disease, cancer, or any other serious disease) and type of clinic attended

^b^Fully-adjusted associations were additionally adjusted for follow-up time

After adjusting for age, sex and age at leaving education, the association between higher nutrition risk scores and lower baseline grip strength, and poorer baseline diet quality, remained. Following further adjustment for number of comorbidities and type of clinic attended, the association between nutrition risk score and baseline prudent diet score remained, but the association with baseline grip strength was attenuated. Our final cross-sectional analyses considered the impact of the inclusion of information about weight loss in the calculation of the nutrition risk score (12 participants lost weight above the DETERMINE threshold, as set out in Table 1, item 9). However, when the recalculated scores were used in age and sex-adjusted models, there was little change in the associations between nutrition risk scores and either grip strength or diet quality (data not shown).

In longitudinal analyses, there was an association between higher baseline nutrition risk score and lower grip strength at follow-up in the sub-group who were reassessed, which remained in the fully-adjusted model (adjusted for sex, age, age left education, number of comorbidities, type of clinic attended and follow-up time). In contrast, there were no associations with the other outcomes assessed at follow-up, in the fully-adjusted analysis.

Cross-sectional associations at follow-up between nutrition risk score and prudent diet score were also assessed; there was an association between higher nutrition risk score at follow-up and lower prudent diet score at follow-up, after adjustment for sex, age and age left education [prudent diet score at follow-up: − 0.16, 95% CI (− 0.28, − 0.04) SD, *p* = 0.009]. However, unlike the baseline cross-sectional association, this was not robust to adjustment in the multivariate model in the sub-group of participants who were followed up [− 0.11, 95% CI (− 0.24, 0.03) SD, *p* = 0.108].

Baseline nutrition risk score was related to greater risk of mortality [unadjusted hazard ratio per unit increase in score: 1.29 (1.01, 1.63), *p* = 0.039] during the follow-up period. However, this association was not robust to adjustment for the effects of sex and age [hazard ratio: 1.08 (0.83, 1.40), *p* = 0.569].

Our final analyses used ROC-curves to evaluate the predictive ability of the nutrition risk score to identify individuals with low grip strength and poor diet quality, both at baseline and at follow-up. There were no significant differences in the area under curve (AUC) when comparing models with sex and age as predictors and those additionally including nutrition risk scores (*p* > 0.1 for all comparisons) (data not shown).

## Discussion

In this study we applied a checklist adapted from the DETERMINE nutrition screening tool to identify nutritional risk, and assessed its relationships with diet quality and health outcomes in a community-dwelling group of older adults in the UK. In cross-sectional analyses at baseline, greater nutritional risk was associated with lower grip strength and with poorer diet quality. However, the association with grip strength was attenuated when adjusting for number of comorbidities. We found no associations between nutrition risk scores and reported weight loss or BMI at baseline. In longitudinal analyses, greater nutritional risk at baseline was associated with lower grip strength at follow-up, even after adjustment for possible confounding factors. In contrast, there were no independent associations with diet quality at follow-up. Furthermore, additional analyses suggested no added predictive value of nutritional risk score, when added to models using sex and age as predictors of mortality or in the prediction of low grip strength or poor diet quality.

The importance of nutrition as an influence on health in older age is widely recognised [23, 24]. However, much current research focuses on malnutrition, and less is known about the preceding determinants of trajectories of change in diet in older age, and adverse changes in nutrition that may be happening before there is unintended weight loss or marked falls in body mass [25]. Early identification of nutritional risk, identifying risk before overt malnutrition has developed, should be key to prevention, prompting intervention to improve outcomes [26]. However, current malnutrition screening tools may not be designed to detect early signs of poor nutrition such as declining diet quality. The observed prevalence of poor diet quality in older populations [27–29], together with findings of low nutrient intakes among older adults who are not at risk of malnutrition when screened [5, 6], highlight the need for new screening approaches to identify and quantify that early risk. Although a recent review identified more than 30 malnutrition screening tools [30], surprisingly few have been developed to screen for other aspects of declining nutrition in older populations. There are validated short dietary assessment questionnaires that quantify dietary intake [13, 31, 32], that do not take account of wider influences on diet, such as the contextual and age-related factors that are known to contribute to nutritional risk [24, 33]. Conversely, other screening methods that address some of these wider determinants of poor nutrition, may not quantify nutritional risk [34–36]. There are, therefore, few studies to compare our findings with directly and none, to our knowledge, in the UK. More research is needed into screening tools that could enable the identification of early signs of poor nutrition, particularly in a UK context.

Our study showed that a short set of eight questions, that can be scored easily, yielded a nutrition risk score that was related prospectively to lower grip strength, an important biomarker of morbidity and mortality [2]. Consistent with this, other studies point to the utility of this tool for the prediction of outcomes related to independence and functional capacity. In a study of US older women, higher nutritional risk assessed with DETERMINE was negatively associated with living independently in the community, and it was suggested that this tool could have potential to identify people who might be at increased risk of losing independence [37]. In a study of independent Japanese community-living older adults, high nutrition risk assessed with this tool at baseline was associated with functional decline in both activities of daily living (ADL) and instrumental ADL (IADL) over a 2-year period [38].

However, although the nutrition risk score was associated with diet quality in cross-sectional analyses, we found no prospective association with overall quality of diet over time in the follow-up sub-group. This may be due to changes in diet over the follow-up period, as a result of ageing-related factors, such as bereavement or onset of illness [24, 39], but is also consistent with mixed evidence from other settings using the DETERMINE tool to indicate differences in diet. For example, in the original study higher scores were linked to greater risk of low nutrient intakes and poorer health [7], but in a cross-sectional US study of community-dwelling older women, the DETERMINE checklist did not identify participants with low nutrient intake (those with < 75% of the recommended intake for eight selected nutrients) [40]. Our study did not find an independent association between baseline nutrition risk score and greater risk of mortality, when adjusted for sex and age. Although some studies have not found the DETERMINE tool to be a significant predictor of mortality in older populations [9, 10], in a relatively large study of older US adults (*n* = 978), nutritional risk calculated using this checklist was associated with all-cause hospitalizations, nonsurgical hospitalizations, and mortality, over a follow-up period of 8.5 years [8].

Because part of our aims was to use diet quality and weight loss as outcomes measures in our analyses, we omitted two items of the DETERMINE checklist, thus the scoring of our adapted tool effectively lowered the overall nutrition risk scores of the participants in our study. However, we found a comparable proportion of older adults categorised as being at high nutritional risk using the published thresholds (17% with score ≥ 6) as reported among adults of similar age in the original DETERMINE study (24%) [7] and to another study of older people in the US [mean age 75.3 (6.7 SD) years], where 20.9% of participants were at high nutritional risk [8]. A similar prevalence was also found in an older European population, where 19% of the Danish participants of the SENECA (Survey in Europe of Nutrition in the Elderly, a Concerted Action) study were found to be at high nutritional risk according to the DETERMINE checklist [10]. A recent systematic review, using data from malnutrition screening tools, indicated that up to 23% of older adults in Europe could be at high risk of malnutrition, across all settings. Moreover, it showed that the prevalence of high malnutrition risk among older adults living in the community was 8.5% [41], which is considerably lower than the figures for high nutritional risk assessed using the DETERMINE tool.

The present study has a number of limitations. Firstly, this was a preliminary study to assess the use of a screening method and its potential to detect early nutritional risk. We did not carry out a power calculation in this exploratory study, and the sample size was small, limiting the statistical power of the study. Furthermore, although we were able to follow up the majority (71%) of participants assessed at baseline, prospective data on grip strength and diet quality were only available for a sub-group of participants. Secondly, there were differences in the way that we derived the variables to be scored using the DETERMINE checklist when compared with the original version, and that affected individual scores. However, we think it is unlikely that small differences in scoring method would explain the associations that we observed. Finally, we studied a small group of older men and women, recruited from outpatient clinics, who had on average more than four comorbidities; thus, study participants may not be representative of the wider population of older adults. This has implications for the generalisability of the findings, and particularly for the prevalence of higher nutritional risk we report, which may be higher in this study than in the broader community-living older adult population, which includes older people not attending outpatient clinics and with likely fewer health conditions on average.

This study found cross-sectional associations between higher nutrition risk scores, assessed from a short checklist, and poorer diet quality, both at baseline and at follow-up. This suggests that this screening method might provide useful information at the time of screening. The nutrition risk score was also associated prospectively with lower grip strength; however, its predictive ability of later outcomes was uncertain, as findings suggested no added value to predictions based on age and sex. Further longitudinal research, with larger study populations, is needed to establish the predictive ability of the tool. Further studies are needed to explore its potential to detect nutritional risk in a range of older populations. Early screening may help to address nutritional risk in a timely manner in older adults living in the community.
